# Preoperative Evaluation of Perineural Invasion in Cervical Cancer: Development and Independent Validation of a Novel Predictive Nomogram

**DOI:** 10.3389/fonc.2021.774459

**Published:** 2021-12-23

**Authors:** Ting Wan, Guangyao Cai, Shangbin Gao, Yanling Feng, He Huang, Lili Liu, Jihong Liu

**Affiliations:** ^1^ Department of Gynecologic Oncology, Sun Yat-sen University Cancer Center, State Key Laboratory of Oncology in South China; Collaborative Innovation Center for Cancer Medicine, Guangzhou, China; ^2^ Department of Pathology, Sun Yat-sen University Cancer Center, Guangzhou, China

**Keywords:** perineural invasion, cervical cancer, predictive model, nomogram, biomarker

## Abstract

**Background:**

Perineural invasion (PNI) is associated with a poor prognosis for cervical cancer and influences surgical strategies. However, a preoperative evaluation that can determine PNI in cervical cancer patients is lacking.

**Methods:**

After 1:1 propensity score matching, 162 cervical cancer patients with PNI and 162 cervical cancer patients without PNI were included in the training set. Forty-nine eligible patients were enrolled in the validation set. The PNI-positive and PNI-negative groups were compared. Multivariate logistic regression was performed to build the PNI prediction nomogram.

**Results:**

Age [odds ratio (OR), 1.028; 95% confidence interval (CI), 0.999–1.058], adenocarcinoma (OR, 1.169; 95% CI, 0.675–2.028), tumor size (OR, 1.216; 95% CI, 0.927–1.607), neoadjuvant chemotherapy (OR, 0.544; 95% CI, 0.269–1.083), lymph node enlargement (OR, 1.953; 95% CI, 1.086–3.550), deep stromal invasion (OR, 1.639; 95% CI, 0.977–2.742), and full-layer invasion (OR, 5.119; 95% CI, 2.788–9.799) were integrated in the PNI prediction nomogram based on multivariate logistic regression. The PNI prediction nomogram exhibited satisfactory performance, with areas under the curve of 0.763 (95% CI, 0.712–0.815) for the training set and 0.860 (95% CI, 0.758–0.961) for the validation set. Moreover, after reviewing the pathological slides of patients in the validation set, four patients initially diagnosed as PNI-negative were recognized as PNI-positive. All these four patients with false-negative PNI were correctly predicted to be PNI-positive (predicted *p* > 0.5) by the nomogram, which improved the PNI detection rate.

**Conclusion:**

The nomogram has potential to assist clinicians when evaluating the PNI status, reduce misdiagnosis, and optimize surgical strategies for patients with cervical cancer.

## Introduction

Cervical cancer is the fourth most common cancer among women worldwide (1). Radical hysterectomy (RH) is a conventional treatment for early-stage cervical cancer that has the advantages of maintaining both ovarian function and sexual function compared with radiotherapy (2, 3). However, extensive parametrial resection during surgery has been proven to cause postoperative pelvic problems, including bladder, sexual, and colorectal dysfunction, which negatively influence quality of life (4). Nerve-sparing radical hysterectomy (NSRH), which was also known as Type C1 radical hysterectomy according to Querleu-Morrow classification to avoid these adverse effects by preserving the pelvic autonomic nerves, has been applied maturely (5). However, controversy still exists regarding the preoperative indications for NSRH. Recent studies have found that dissemination along nerves is considered an independent route for cancer spread (6, 7). NSRH may preserve not only the nerves but also the cancer cells invading the nerves. Perineural invasion (PNI) is reportedly associated with multiple high-risk factors (8, 9) and poor outcomes during early-stage cervical cancer (10, 11). PNI is relatively common in cervical cancer and may be underestimated. Pathological examinations have shown that 7.1%–35.1% of patients with early-stage cervical cancer have PNI (8–14). Therefore, preoperative diagnosis of PNI could help identify populations who would benefit from NSRH.

Unfortunately, it is not easy to identify signs of PNI before surgery. Although it has been reported that some patients with cervical cancer and PNI have different degrees of pelvic pain, this symptom was rare and not sufficiently typical (15). Researchers have examined PNI diagnosis in other types of cancer, such as colon, prostate, and pancreatic cancers, to distinguish PNI with magnetic resonance imaging (MRI) or positron emission tomography and computed tomography (CT) (16, 17). Nevertheless, few studies have investigated preoperative detection of PNI in cervical cancer.

In this study, we aimed to explore the relative clinical and radiological factors of PNI in cervical cancer and develop a predictive nomogram for PNI using preoperative clinical and radiological data.

## Materials and Methods

### Participants

**Figure 1:**
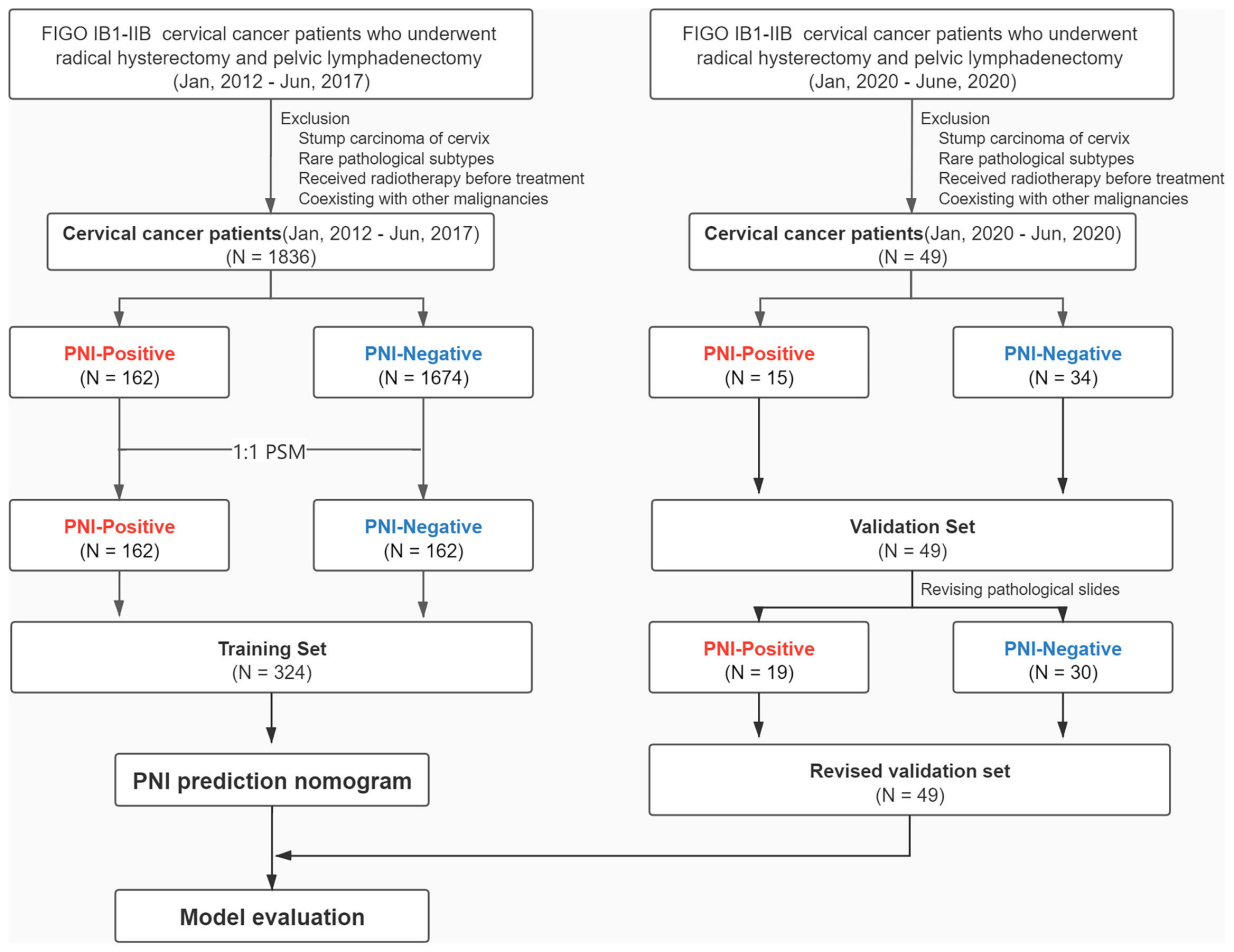
Flowchart of the study design.

We screened 1836 patients diagnosed with FIGO stage IB1–IIB cervical cancer at Sun Yat-sen University Cancer Center who were admitted between January 1, 2012, and June 1, 2017, and underwent standard RH during hospitalization. Patients were excluded if they had any of the following conditions: cervical stump cancer; histological types except squamous carcinoma, adenocarcinoma, or adenosquamous carcinoma; cervical conization or radiotherapy before RH, and a history of other malignant tumors. Patients who had cervical conization before RH were excluded because it was difficult to get all the conization pathological slices to evaluate the PNI status if the conization was done in other hospitals. Additionally, neoadjuvant chemotherapy could be performed only for patients with FIGO stage IB3/IIA2/IIB. A total of 162 cervical cancer patients with PNI (PNI-positive) and 1674 cervical cancer patients without PNI (PNI-negative) were included in the training set. To avoid underestimation of the real incidence of PNI, all pathological slides of 1836 patients were to be re-read by pathologists, but this task was too difficult to complete. Therefore, we applied 1:1 propensity score matching using SPSS (version 23.0) to balance the following important patient characteristics: tumor size, histological type, FIGO stage, differentiation, and preoperative treatment (matching tolerance = 0.01) (18). Eventually, 162 matched pairs of PNI-positive and PNI-negative patients were included in the training set. The validation set comprised 49 eligible patients who were randomly enrolled using the same inclusion and exclusion criteria and who were admitted between January 1, 2020, and June 1, 2020. The study design is illustrated in **Figure 1**.

### Data Collection

In our published data, we found PNI in cervical cancer was associated with deep stromal of cervical canal invasion, Lymph node invasion, and positive margin (18). This result inspired us that the occurrence of PNI should be associated with risk factors for cervical cancer. Also, we considered factors proven to be associated with PNI in previous studies (8, 19, 20). Therefore, in this study, we collected preoperative clinical and radiological data from the electronic health records accordingly. Clinical data included age, FIGO stage, histological type (determined using thinprep cytology test or cervical biopsy results), degree of differentiation (determined using cervical biopsy results), and neoadjuvant chemotherapy (NACT). Radiological data included tumor size, lower uterine segment invasion, deep stromal invasion (DSI), full-layer invasion (FLI), and lymph node enlargement (LNE), all of which were indicated by radiology before all anti-tumor treatment. Senior radiologists in gynecological oncology subspeciality from the radiology department of Sun Yat-sen University Cancer Center confirmed the quality and reports of MRI or CT for every patient. Trained researchers entered and double-checked the data independently.

To diagnose PNI, surgical specimens were fixed with 10% neutral formaldehyde fixation solution, embedded in paraffin, cut into 4-μm-thick sections, and stained with hematoxylin and eosin. Patients were classified as PNI-positive if the microscopic examination found that cancer cells infiltrated any layer of nerve fibers (including the epineurium, perineurium, and endoneurium) or surrounded more than 33% of the outer diameter of nerves. If hematoxylin and eosin staining could not verify PNI, then immunohistochemical staining of the nerve bundle S-100 was used to identify the nerves (21). The FIGO staging of all patients was performed according to the 2018 FIGO Staging guidelines (22). The histological type was obtained from the cervical biopsy results and categorized according to whether the adenocarcinoma component was present. The degree of differentiation was also determined using cervical biopsy results and classified as good, moderate, or poor. Lymph vascular space invasion (LVSI) was defined as the presence of tumor cells in a space lined by endothelial cells outside the immediate invasive border on postoperative pathological examination; therefore, we collected its data and used it as a baseline characteristic to observe but not as a predictive variable. Lower uterine segment invasion was defined as a cervical tumor extending above the uterine isthmus on preoperative CT or MRI. DSI was defined as a cervical tumor invading more than half of the cervical canal from the external cervical orifice to the cervical isthmus and more than half the thickness of the cervical transverse muscle. FLI was defined as cervical mass invasion into the epigastric layer of the cervix. LNE was defined as the pelvic lymph nodes with the short axis diameter ≥5 and ≤15 mm on CT or MRI (23–25) (i.e., lymph node metastasis that was suspected but not confirmed was included in the study). Para-aortic lymph nodes were not evaluated here because patients with suspicious enlarged para-aortic lymph nodes didn’t receive RH in our center, which were considered as distant metastasis of cervical cancer previously (26, 27).

### Model Development and Evaluation

#### Nomogram Development

First, a logistic regression model was constructed using the Stats package of R language (Version 4. 0. 1, Vienna, Austria) and variables were screened using stepwise regression with the CAR package. We included variables in the logistic regression analysis based on previous studies and clinical consensus (28). Then, we constructed the PNI prediction nomogram based on the logistic regression model with the regplot package. Each variable was given a score based on the point scale of the nomogram according to the coefficients in the logistic regression equation. By summing the total scores, we were able to estimate the probability of PNI for cervical cancer patients before surgery. Probability less than 50% was considered low risk for PNI, whereas probability more than 50% was considered high risk for PNI. The higher the total score, the higher the risk of PNI.

#### Evaluation of the Model

The nomogram was validated internally for the training group and externally for the validation group. We evaluated the predictive performance of the nomogram using the receiver-operating characteristics (ROC) curve, calibration curve, and performance metrics including the area under the ROC curve (AUC), accuracy, sensitivity, specificity, positive predictive value, negative predictive value, F1 score, and Cohen’s kappa coefficient (kappa) using R packages pROC, RMS, and caret.

### Statistical Analysis

The median value (interquartile range) and frequency (%) were used to express continuous and categorical variables, respectively. All continuous variables were compared between groups using the Mann–Whitney U test. All categorical variables were compared using the chi-square test or Fisher’s exact test, as appropriate. The odds ratio (OR) and corresponding 95% confidence interval (95% CI) from the logistic regression were calculated to assess the strength of association between clinical or radiological factors and the occurrence of PNI using R package stats. The significance level (*p*) was set at <0.05 (two-sided *p* value).

### Ethical Consideration

This study was approved by the Ethics Committee and Institutional Review Board of the Sun Yat-sen University Cancer Center (Guangzhou, China). All case data were anonymized, and the Institutional Review Board waived the requirement for written informed consent because it did not involve breach of patient privacy.

## Results

**Table 1** lists the clinical characteristics of the training set. Patients who were older [PNI-positive vs. PNI-negative: 51.5 years (interquartile range, 45.25–57) vs. 49 years (interquartile range, 41.25–55); *p* = 0.006], had LNE (35.2% vs. 17.3%; *p* < 0.001), had DSI (66.0% vs. 39.5%]; *p* < 0.001), or had FLI (45.1% vs. 10.5%; *p* < 0.001) were significantly more likely to have PNI. In addition to matched factors, LVSI (33.3% vs. 31.5%; *p* = 0.812) and lower uterine segment invasion (23.5% vs. 16.0%; *p* = 0.125) were not significantly different between the PNI-positive and PNI-negative groups.

**Table 1:** Baseline characteristics of the individuals in the training set.

		PNI-negative	PNI-positive	*p value*
		n=162	n=162	
**Age (years)**		49 [41.25, 55]	51.5 [45.25, 57]	0.006
**FIGO stage (%)**	IB1	38 (23.5%)	41 (25.3%)	0.996
	IB2	14 (8.6%)	13 (8.0%)	
	IIA1	60 (37.0%)	59 (36.4%)	
	IIA2	27 (16.7%)	26 (16.0%)	
	IIB	23 (14.2%)	23 (14.2%)	
**Adenocarcinoma (%)**	No	118 (72.8%)	116 (71.6%)	0.901
	Yes	44 (27.2%)	46 (28.4%)	
**Differentiation (%)**	Good	6 (3.7%)	8 (4.9%)	0.858
	Moderate	63 (38.9%)	63 (38.9%)	
	Poor	93 (57.4%)	91 (56.2%)	
**LVSI (%)**	No	111 (68.5%)	108 (66.7%)	0.812
	Yes	51 (31.5%)	54 (33.3%)	
**Tumor size (cm)**		4.0 [3.0, 4.5]	3.9 [3.0, 4.65]	0.929
**LNE (%)**	No	134 (82.7%)	105 (64.8%)	<0.001
	Yes	28 (17.3%)	57 (35.2%)	
**LUSI (%)**	No	136 (84.0%)	124 (76.5%)	0.125
	Yes	26 (16.0%)	38 (23.5%)	
**DSI (%)**	No	98 (60.5%)	55 (34.0%)	<0.001
	Yes	64 (39.5%)	107 (66.0%)	
**FLI (%)**	No	145 (89.5%)	89 (54.9%)	<0.001
	Yes	17 (10.5%)	73 (45.1%)	
**NACT (%)**	No	98 (60.5%)	111 (68.5%)	0.164
	Yes	64 (39.5%)	51 (31.5%)	

Continuous variables are presented as median (interquartile ranges [IQR]) while categorical variables as counts and percentages (%). PNI, perineural invasion; FIGO stage, International Federation of Gynecology and Obstetrics stage; LVSI, lymph vascular space invasion; LNE, lymph node enlargement; LUSI, lower uterine segment invasion; DSI, deep stromal invasion; FLI, full-layer invasion; NACT, neoadjuvant chemotherapy.

**Figure 2:**
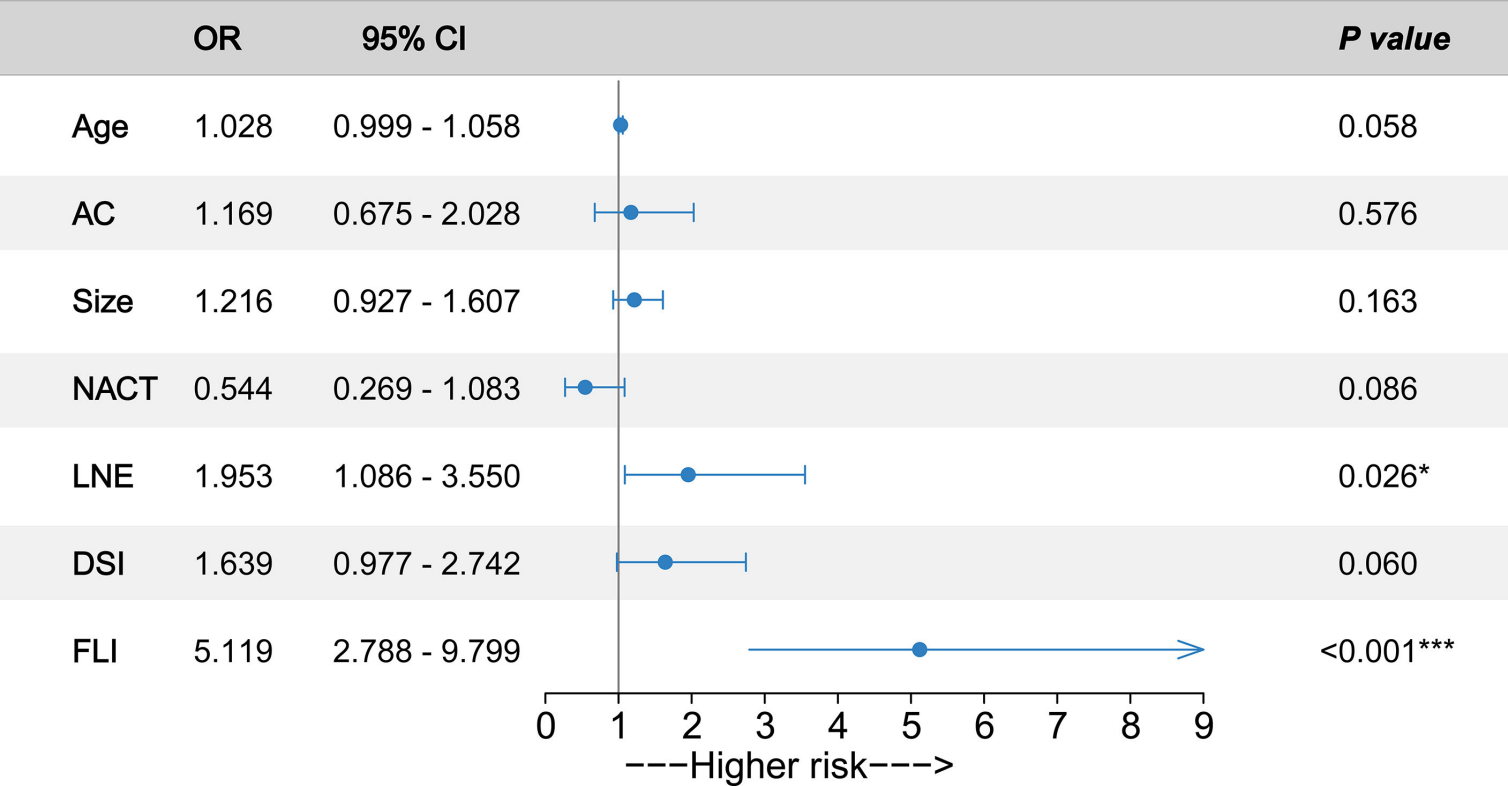
Odds ratios (ORs) of predictors associated with perineural invasion (PNI) occurrence. Forrest plot with ORs and 95% confidence intervals (CIs) according to the multivariate logistic regression analysis. The circles represent the ORs of the predictors. Whiskers represent 95% CI. AC, adenocarcinoma; Size, tumor size; NACT, neoadjuvant chemotherapy; LNE, lymph node enlargement; DSI, deep stromal invasion; FLI, full-layer invasion. ***p < 0.001. *p < 0.05.

Next, we conducted a multivariate logistic regression analysis to predict the PNI status of cervical cancer patients. The pathological diagnosis of PNI was identified as an outcome variable. Backward stepwise selection with the Akaike information criterion was performed for predictor variable screening to build the final multivariate logistic regression model. In particular, we included adenocarcinoma, tumor size, and NACT as predictive variables in the final model because prior studies have shown that these variables are associated with PNI (10, 29). Finally, seven predictor variables were integrated into the multivariate logistic regression model for PNI prediction (**Figure 2**). According to the model parameters, FLI (OR, 5.119; 95% CI, 2.788–9.799; *p* < 0.001) and LNE (OR, 1.953; 95% CI, 1.086–3.550; *p* = 0.026) were significantly associated with an increased risk of PNI for cervical cancer patients. Age (OR, 1.028; 95% CI, 0.999–1.058; *p* = 0.058) and DSI (OR, 1.639; 95% CI, 0.977–2.742; *p* = 0.060) were also associated with the higher risk of PNI (*p* values were near the significance threshold of 0.05).

**Figure 3:**
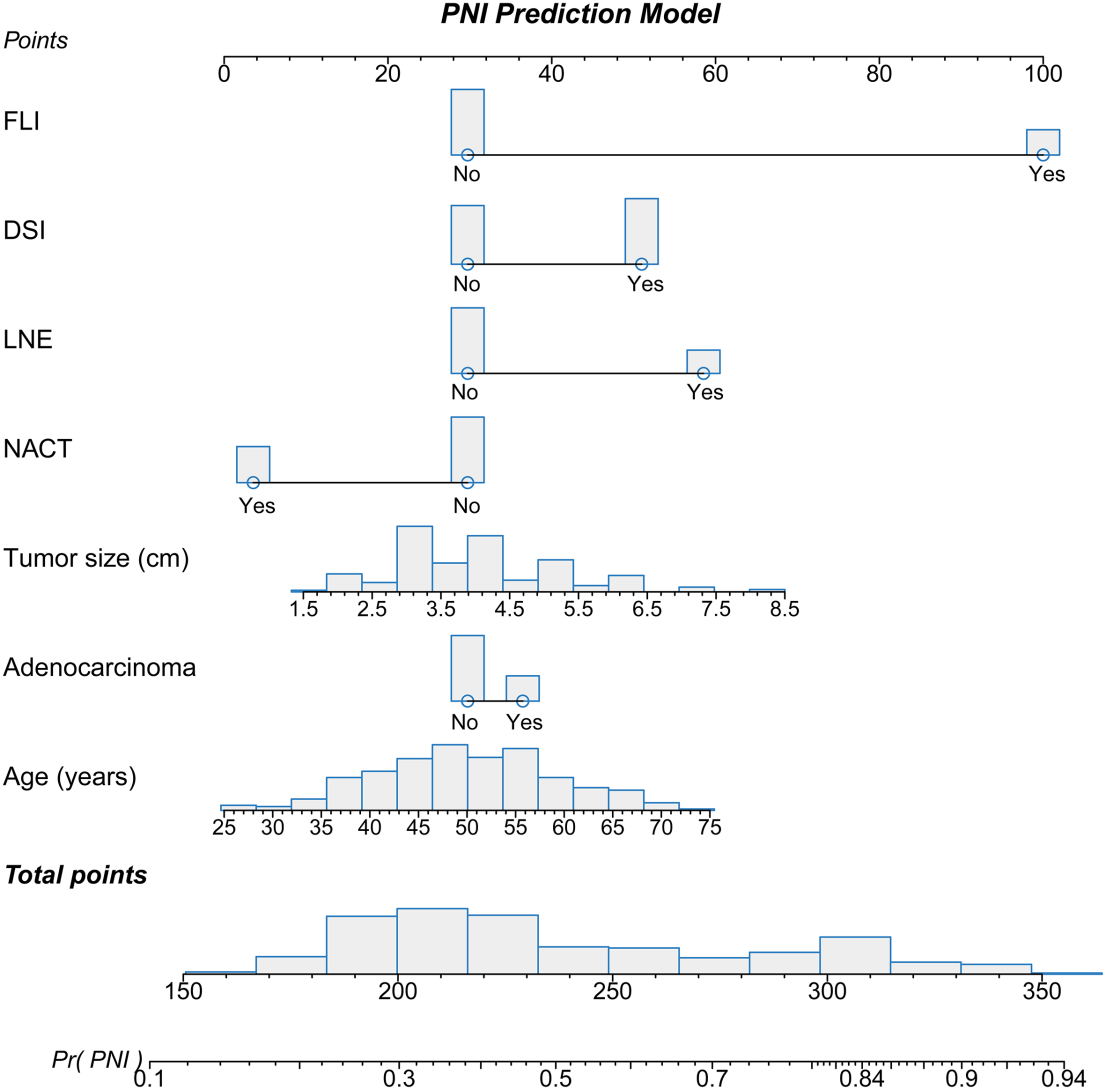
Nomogram used to predict the probability of perineural invasion (PNI) in cervical cancer patients based on multivariate logistic regression. Each variable was given a point on the scale that was correlated with the odds ratio. After summing all points of the variables, we obtained the total point score for each patient. The total point score was used to determine the probability of PNI for cervical cancer patients. The distribution of each variable is presented as a bar graph. The point assignments are presented in **Table S1**.

**Figure 4:**
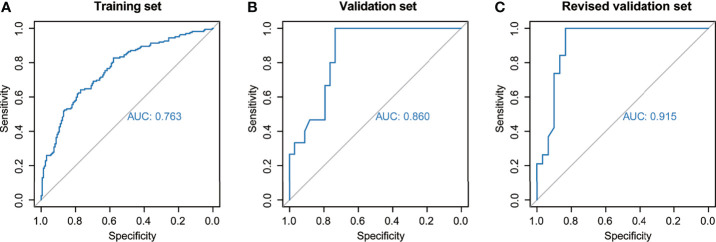
Predictive performance of the model across different sets. The area under the receiver-operating characteristic curve was used to assess perineural invasion prediction using the nomogram for the **(A)** training set, **(B)** validation set, and **(C)** revised validation set.

The nomogram was established based on the final logistic regression model (**Figure 3**). The score assignment of the predictor variables is shown in **Table S1**. The nomogram achieved an AUC of 0.763 (95% CI, 0.712–0.815) for the training set and 0.860 (95% CI, 0.758–0.961) for the validation set (**Figures 4A, B**). The performance matrix, including sensitivity, specificity, positive predictive value, negative predictive value, accuracy, F1 scores, and kappa values, of the two sets is shown in **Table 2**. The calibration curves of the model for the two sets (**Figures S1**, **S2**) indicated that the PNI prediction model displayed mean absolute scores of 0.021 for the training set and 0.12 for the validation set, which meaning that the prediction probability of this model is close to the actual probability.

**Table 2:** Performance of the nomogram in predicting PNI in different groups.

	Training Set		Validation Set		Revised Validation Set	
	Value	95% CI	Value	95% CI	Value	95% CI
**AUC**	0.763	0.712 - 0.815	0.860	0.758 - 0.961	0.915	0.832 - 0.998
**Sensitivity**	59.9%	51.9% - 67.5%	100%	78.2% - 100%	100%	82.4% - 100%
**Specificity**	79.6%	72.6% - 85.5%	64.7%	46.5% - 80.3%	73.3%	54.1% - 87.7%
**PPV**	74.6%	67.9% - 80.3%	55.6%	44.2% - 66.3%	70.4%	56.8% - 81.1%
**NPV**	66.5%	61.8% - 70.9%	100%	NA	100%	NA
**Accuracy**	69.8%	64.4% - 74.7%	75.5%	61.1% - 86.7%	83.7%	70.3% - 92.7%
**F1**	0.664		0.714		0.826	
**Kappa**	0.395		0.529		0.681	

AUC, area under the receiver operating characteristics curve; PPV, positive predictive value; NPV, negative predictive value; 95% CI, 95% confidence interval; NA, not available.

**Figure 5:**
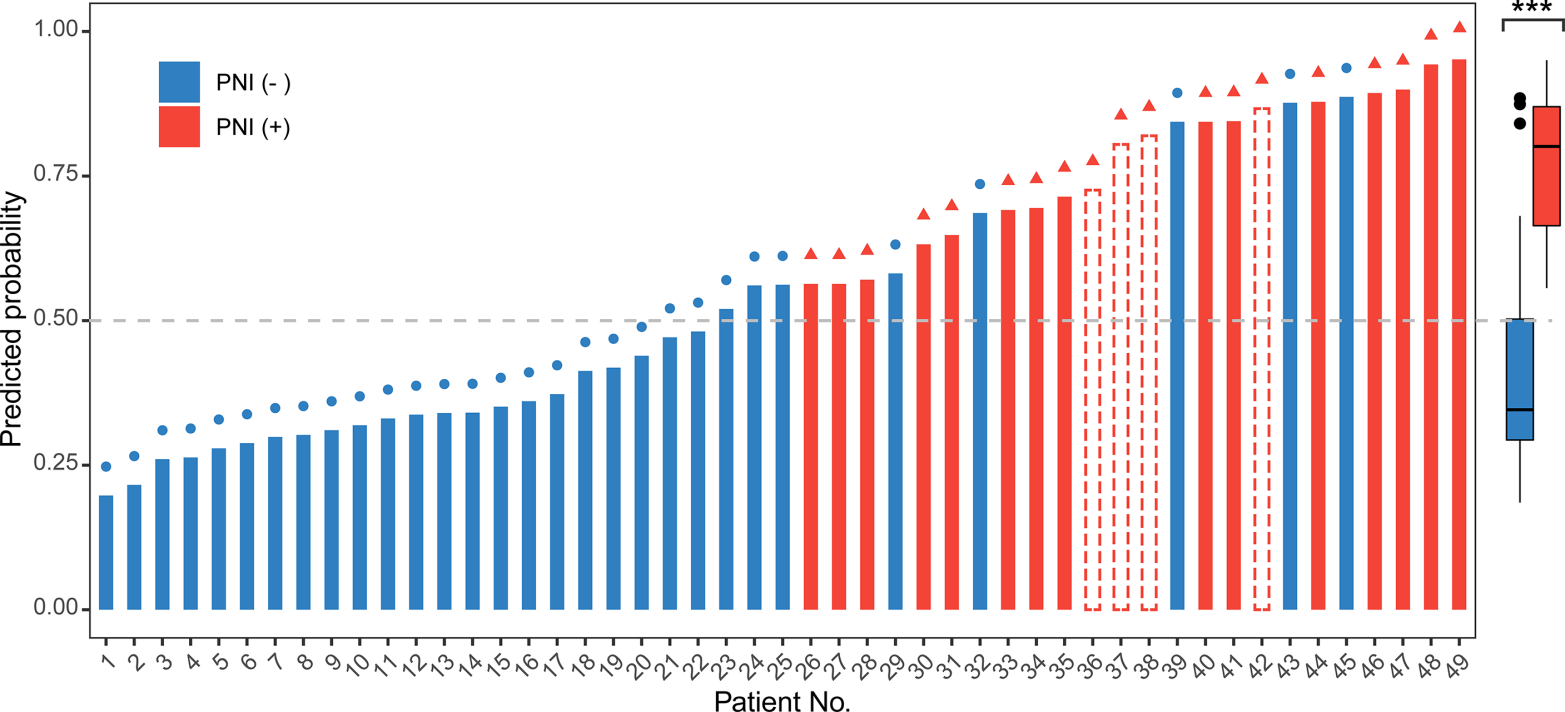
Use of the nomogram to predict the probability of perineural invasion (PNI) occurrence for all 49 patients in the validation set. The predicted probability of PNI more than 0.5 (gray dashed line) was regarded as PNI-positive. In the left graph, the color of the bar represents the real status of PNI according to the pathological examination. The red bar represents PNI-positive, the blue bar represents PNI-negative, and the bar with the red dashed border represents PNI-positive patients who were misdiagnosed as PNI-negative before revision but were correctly predicted to be PNI-positive by the nomogram. The symbols on the top of each bar indicate the final pathological diagnosis of the PNI status after revision. A red triangle at the end of a line indicates that the patient had PNI. A blue circle indicates that the patient did not have PNI. The right box plot shows the distribution of the predicted probability of PNI for PNI-positive and PNI-negative patients included in the revised validation set. The center line represents the median probability of PNI in the different groups. Box limits represent the upper and lower quartiles. Whiskers represent a 1.5-times interquartile range. The black points represent the outliers. The Wilcoxon test was performed for the univariate comparison between groups. A two-tailed *p*-value of <0.05 was considered statistically significant. ***p < 0.001.

Moreover, we invited experienced pathology specialists on gynecological oncology to review the pathological slides of patients in the validation set. Four patients who had been initially diagnosed as PNI-negative were recognized as PNI-positive, whereas the PNI diagnoses of the other patients were consistent with the original diagnoses. The baseline characteristics of the original and revised validation sets are shown in **Table 3**. After revision, the performance of the model for the validation set markedly improved (**Figure 4C**, **Table 2** and **Figure S3**). The AUC of the revised validation set was 0.915 (95% CI, 0.832–0.998) (**Figure 4C** and **Table 2**). The specificity of the revised validation set (73.3%; 95% CI, 54.1%–87.7%) increased compared with that of the original validation set (64.7%; 95% CI, 46.5%–80.3%), and the sensitivity remained 100% after revision (**Table 2**). Additionally, the calibration curve showed better agreement after revision; the mean absolute score improved from 0.12 to 0.095 (**Figure S3**), indicating that the PNI prediction model could help reduce the diagnosis of false-negative PNI for cervical cancer patients. The predicted probability of PNI for PNI-positive patients was significantly greater than that for PNI-negative patients, thereby showing good discriminability (**Figure 5**).

**Table 3:** The baseline characteristics of the original and revised validation sets.

		Validation Set			Revised Validation Set		
		PNI-Negative	PNI-Positive	*p value*	PNI-Negative	PNI-Positive	*p value*
		n=34	n=15		n=30	n=19	
**Age (years)**		53 [46.5, 59.5]	51 [41, 62.5]	0.983	52.5 [46, 58]	53 [44, 62]	0.572
**FIGO stage (%)**	IB1	18 (52.9)	6 (40.0)	0.599	18 (60.0%)	6 (31.6%)	0.100
	IB2	16 (47.1)	9 (60.0)		12 (40.0%)	13 (68.4%)	
	IIA1	0 (0.0%)	0 (0.0%)		0 (0.0%)	0 (0.0%)	
	IIA2	0 (0.0%)	0 (0.0%)		0 (0.0%)	0 (0.0%)	
	IIB	0 (0.0%)	0 (0.0%)		0 (0.0%)	0 (0.0%)	
**Adenocarcinoma (%)**	No	23 (67.6)	10 (66.7)	1.000	19 (63.3%)	14 (73.7%)	0.660
	Yes	11 (32.4)	5 (33.3)		11 (36.7%)	5 (26.3%)	
**Tumor size (cm)**		3.5 [2.55, 4.5]	4.0 [3.5, 4.75]	0.256	3.5 [2.5, 4.42]	4.2 [3.5, 5.0]	0.041
**LNE (%)**	No	29 (85.3)	3 (20.0)	<0.001	25 (83.3%)	7 (36.8%)	0.003
	Yes	5 (14.7)	12 (80.0)		5 (16.7%)	12 (63.2%)	
**LUSI (%)**	No	29 (85.3)	4 (26.7)	<0.001	26 (86.7%)	7 (36.8%)	0.001
	Yes	5 (14.7)	11 (73.3)		4 (13.3%)	12 (63.2%)	
**DSI (%)**	No	29 (85.3)	1 (6.7)	<0.001	26 (86.7%)	4 (21.1%)	<0.001
	Yes	5 (14.7)	14 (93.3)		4 (13.3%)	15 (78.9%)	
**FLI (%)**	No	26 (76.5)	7 (46.7)	0.085	26 (86.7)	7 (36.8)	0.001
	Yes	8 (23.5)	8 (53.3)		4 (13.3)	12 (63.2)	
**NACT (%)**	No	34 (100.0)	15 (100.0)	1.000	30 (100.0%)	19 (100.0%)	1.000
	Yes	0 (0.0%)	0 (0.0%)		0 (0.0%)	0 (0.0%)	

Continuous variables are presented as median [interquartile ranges (IQR)] while categorical variables as counts and percentages (%). PNI, perineural invasion; FIGO stage, International Federation of Gynecology and Obstetrics stage; LNE, lymph node enlargement; LUSI, lower uterine segment invasion; DSI, deep stromal invasion; FLI, full-layer invasion; NACT, neoadjuvant chemotherapy.

## Discussion

We conducted a large-scale retrospective study in China to explore preoperative clinical and radiological factors associated with PNI in cervical cancer patients and to establish a PNI prediction nomogram for cervical cancer based on a multivariate logistic regression analysis including training and validation sets. Our study expands the literature regarding PNI-associated clinical characteristics and provides a feasible model for the preoperative evaluation of PNI.

In this study, we analyzed ten clinical and radiological factors according to previous researches. Seven were finally included in the final prediction nomogram. Based on the consensus, FLI and DSI indicate more locally invasive cancer (30). During this study, FLI and DSI were important predictors of PNI. Therefore, it is reasonable to hypothesize that the complex interactions among neurogenic molecules, cancer cells, and the cancer microenvironment contribute to the local spread of cancer. Adenocarcinoma, LNE, and tumor size not only were risk factors for cancer progression in cervical cancer (19) but also were associated with the occurrence of PNI in previous studies (20). NACT could kill cancer cells in the body and reduce the detection rate of PNI in later surgical specimens. The inclusion of these factors increased interpretability of the prediction model. Intriguingly, no significant difference in LVSI was found between the PNI-positive and PNI-negative groups (**Table 1**). This provided a glimpse of neural invasion as a potential independent metastasis pathway different from lymphatic metastasis, suggesting that more attention should be focused on PNI during the comprehensive evaluation of cervical cancer.

The preoperative prediction of PNI in cervical cancer has important clinical implications. PNI is a sign of tumor metastasis and invasion (31). PNI in cervical cancer is significantly correlated with high risk and a poorer prognosis (8, 9, 11, 32, 33). A recent study suggested that microenvironment remodulation has an important role in PNI occurrence. Cross-talk among neural cells, supporting cells, and malignant tumor cells gradually leads to changes in and migration of the perineural matrix (31, 34). Therefore, PNI prediction can contribute to blocking cancer progression and improving patient survival (35, 36).

PNI may help optimize preoperative treatment decisions for cervical cancer patients. NSRH has been a treatment choice for patients with early-stage cervical cancer resulting in a higher quality of life than conventional RH. However, the population in which it is applicable remains controversial because of concerns regarding the safety of conserving invaded nerves. The removal of peripheral nerves has been shown to inhibit tumor invasion and metastasis associated with other malignancies. The formation of autonomic nerve fibers in the prostate has been reported to modulate the development and spread of prostate cancer in a mouse model, and the densities of sympathetic and parasympathetic nerve fibers in the tumor and surrounding normal tissues were correlated with adverse clinical outcomes during a retrospective blind analysis of prostate adenocarcinoma samples (37). Surgical denervation and drug denervation can significantly reduce the incidence and progression of tumors in animal models of gastric cancer (38). In a very large series from Europe, the rate of postoperative adjuvant therapy was 48% after radical hysterectomy for early-stage cervical cancer (5), but the adverse prognosis caused by PNI may not be completely eliminated by adjuvant therapy. A systematic review of cervical cancer found that more deaths were observed in the NSRH group than in the RH group (two in the NSRH group vs. zero in the RH group); however, all included patients had received standard postoperative adjuvant therapy (39). Since the presence of PNI was associated with the optimal resection of tumors during NSRH, preoperative PNI prediction might help to identify which populations could obtain maximum benefits from NSRH without compromising oncologic safety.

Recently, some studies have focused on preoperatively predicting PNI. Liu et al. constructed a nomogram including carcinoembryonic antigen levels, tumor size, Lauren classification, radiological stage, and lymph node metastasis to predict the PNI status with advanced gastric cancer (AUC of 0.935 for the internal validation set and AUC of 0.828 for the external validation set) (40). PNI prediction models with clinical factors have also been reported for colorectal cancer, head and neck squamous cancer, oral cancer, and pancreatic cancer (41–45). These findings suggest that using clinical pathological features to build a PNI prediction model is feasible. However, few researchers have investigated the prediction of PNI in cervical cancer. During this study, we built an effective PNI prediction nomogram for cervical cancer based on preoperative clinical and radiological factors. The AUC, sensitivity, and specificity were 0.763, 59.9%, and 79.6%, respectively, for the training set and 0.915, 100%, and 73.3%, respectively, for the revised validation set, thereby indicating its satisfactory prediction performance. We found that this prediction model could help identify patients with false-negative PNI, which is valuable to improving the diagnosis rate of PNI and helping unexperienced pathologists at smaller hospitals.

The two prominent strengths of this study are the large volume of PNI-positive cervical cancer patients and the comparable population with a different PNI status after propensity score matching, which allowed for a comprehensive analysis of multiple clinical and radiological factors. However, our study has some limitations. First, it is a single-center retrospective study; therefore, only variables already captured could be used for analysis. Second, we did not adjust for all possible confounders. Lastly, the generalizability of the nomogram is limited to the size of our external validation set. Larger-scale, multicenter investigations should be performed at different hospitals and in different regions to verify the findings of this study before our nomogram can be applied in practice.

## Conclusions

This study explored factors correlated with the occurrence of PNI in cervical cancer. We constructed a feasible nomogram to predict PNI occurrence. This nomogram has the potential to assist clinicians when evaluating the PNI status and reduce the misdiagnosis of PNI preoperatively, thus optimizing treatment decisions for cervical cancer patients.

## Data Availability Statement

The raw data supporting the conclusions of this article will be made available by the authors, without undue reservation.

## Ethics Statement

The studies involving human participants were reviewed and approved by Ethics Committee and Institutional Review Board of the Sun Yat-sen University Cancer Center (Guangzhou, China). The ethics committee waived the requirement of written informed consent for participation.

## Author Contributions

JL and TW designed the study. TW and GC performed the analysis, interpreted the data, and wrote the paper. SG collected patient samples and clinical data. YF and HH helped analyze the data. JL advised on the conception and design of the study. All authors vouch for the respective data and analysis, approved the final version, and agreed to publish the manuscript.

## Funding

This work received support from the Collaborative Innovation Foundation of Guangzhou, China (No. 2015082020264).

## Conflict of Interest

The authors declare that the research was conducted in the absence of any commercial or financial relationships that could be construed as a potential conflict of interest.

## Publisher’s Note

All claims expressed in this article are solely those of the authors and do not necessarily represent those of their affiliated organizations, or those of the publisher, the editors and the reviewers. Any product that may be evaluated in this article, or claim that may be made by its manufacturer, is not guaranteed or endorsed by the publisher.

## Supplementary Material

Supplementary Figure 1 Calibration curves of the model for the training set.

Supplementary Figure 2 Calibration curves of the model for the validation set.

Supplementary Figure 3 Calibration curves of the model for the revised validation set.

Supplementary Table 1 The point assignments of the nomogram.

## Abbreviations

PNI, perineural invasion; NACT, neoadjuvant chemotherapy; LNE, lymph node enlargement; DSI, deep stromal invasion; FLI, full-layer invasion; RH, radical hysterectomy; NSRH, nerve-sparing radical hysterectomy; MRI: magnetic resonance imaging; LVSI, lymph vascular space invasion; LUSI, lower uterine segment invasion; ROC curve, receiver-operating characteristics curve; AUC, area under the ROC curve; PPV, positive predictive value; NPV, negative predictive value; OR, odds ratio; 95% CI, 95% confidence interval; IQR, interquartile range; CT, computed tomography.
