# Supplementary figures and images for: Preoperative Evaluation of Perineural Invasion in Cervical Cancer: Development and Independent Validation of a Novel Predictive Nomogram
Source: Front Oncol. 2021 Dec 23;11:774459. doi: 10.3389/fonc.2021.774459 (PMC8733474; doi:10.3389/fonc.2021.774459)

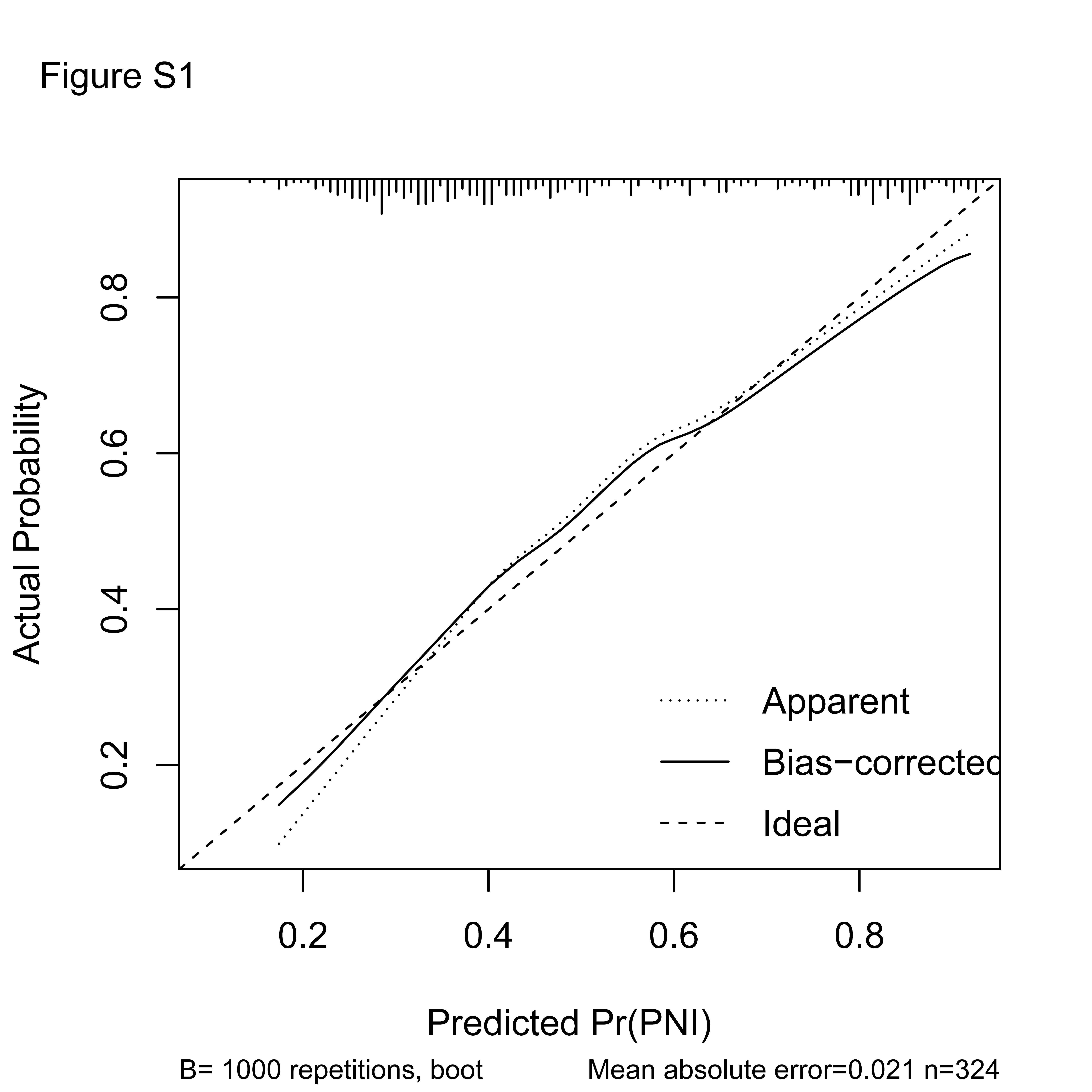

Supplement: Supplementary Figure 1 — Calibration curves of the model for the training set. [file Image_1.tif]

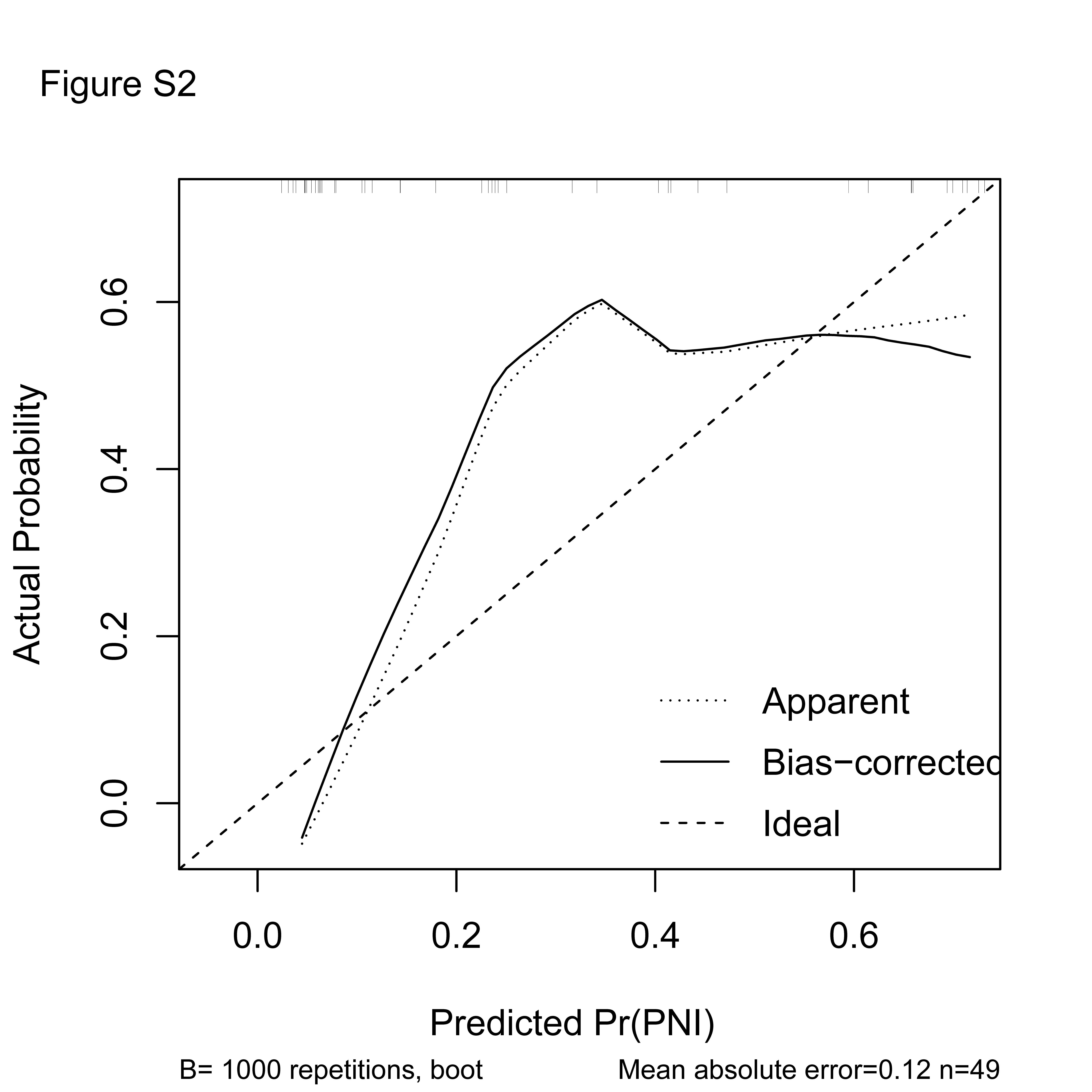

Supplement: Supplementary Figure 2 — Calibration curves of the model for the validation set. [file Image_2.tif]

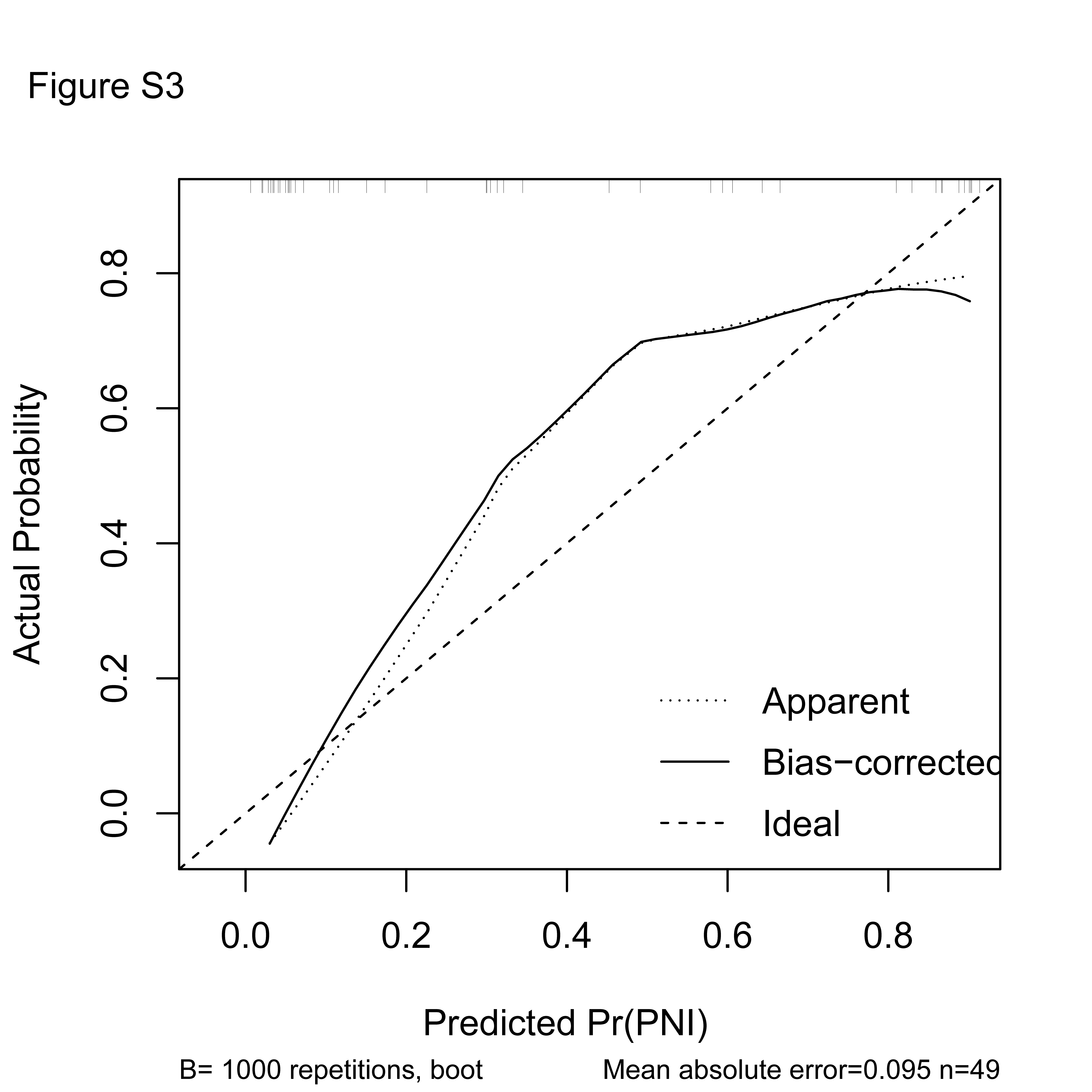

Supplement: Supplementary Figure 3 — Calibration curves of the model for the revised validation set. [file Image_3.tif]
